# The Effects of Vitamin B1 on Ameliorating the Premenstrual Syndrome Symptoms

**DOI:** 10.5539/gjhs.v6n6p144

**Published:** 2014-07-29

**Authors:** Sareh Abdollahifard, Afifeh Rahmanian Koshkaki, Reza Moazamiyanfar

**Affiliations:** 1 Department of Nursing and Midwifery, Jahrom University of Medical Sciences, Jahrom, Iran

**Keywords:** premenstrual syndrome, vitamin B1, placebo

## Abstract

**Background and Objective:**

The premenstrual syndrome (PMS) is a series of physical, mental, and behavioral symptoms with various severities, and disturbs social and personal relationships. The syndrome appears during luteal phase of the menstrual cycle and is a common disorder of reproductive age. Different treatments have been introduced for the syndrome due to its unknown complicated causes. Vitamin B1 (Thiamin) may reduce symptoms of the syndrome through affecting the performance of coenzymes in the metabolism of carbohydrates and main branch of amino acid that plays an important role in appearance of physical and mental symptoms of the PMS. Vitamin B1 is the first water-soluble discovered vitamin. As it is effective in neural activity and muscle tonus in different body activities, including hematopoiesis, metabolism of carbohydrates, activities of the central nervous system and neuromuscular system, etc., it can be effective in this dysmenorrhea that is a disorder resulting from uterine muscular contraction. There are no enough studies and research on the effect of vitamin B1 on the symptoms of PMS, therefore, this study was conducted to determine the effect of vitamin B1 on the symptoms of PMS in students residing at dormitories of Jahrom University of Medical Sciences in 2013.

**Methods:**

In this double-blind placebo-controlled clinical trial, 80 students with PMS residing at dormitories of Jahrom University of Medical Sciences were divided randomly into two groups, vitamin B1 and placebo. The severity of the symptoms of PMS in two cycles, before the intervention and during the intervention, was recorded by the students. The data were collected using an information collection form, PMS provisional diagnosis form, daily status record form, Beck Depression Inventory. The data were analyzed using descriptive and inferential statistics.

**Results:**

There was no significant difference among the studied variables in terms of confounding variables. The comparison of vitamin B1 group before the intervention with that after the intervention showed that vitamin B1 reduced mean mental (35.08%) and physical (21.2%) symptoms significantly (P < 0.0001). Moreover, there was a significant difference between vitamin B1 and placebo groups in terms of mean mental and physical symptoms, as mean symptoms in vitamin B1 group was significantly lower than that in the placebo group (P < 0.0001).

**Conclusion:**

It seems that vitamin B1 is effective in recovery of mental and physical symptoms of PMS. Therefore, this vitamin can be used to reach a major goal of midwifery, that is, reduction of symptom severity of PMS, without any side effects.

## 1. Introduction

The premenstrual syndrome (PMS) is a common disorder in the reproductive age and appears in 85-90% of women in the reproductive age with various degrees (Abdollahifard et al., 2013; Bakhshani et al., 2011; Gehlert et al., 2009; Pourmohsen et al., 2010). The disorder consists of a series of physical, emotional, and behavioral symptoms appearing regularly and periodically in relation to luteal phase of the menstrual cycle in most cycles (Gehlert et al., 2009; Abdollahifard et al., 2012). Symptoms of the PMS are as follows: fatigue, irritability, edema, anxiety, tension, mastalgia (breast tenderness), mood swings, depression, acne, increased appetite, crying for no particular reason, headaches, forgetfulness, gastrointestinal symptoms, poor concentration, flushing, flatulence, limb edema, and dizziness (Speroff & Fritz, 2011; Berek, 2011). The disorder may cause marital relation disruption, mother-child problems, social isolation, decreased attention, increased psychosomatic symptoms, and even suicide and legal problems (Abdollahifard et al., 2013; Braverman, 2007). Although PMS is a common condition in the world, it is difficult to measure its actual prevalence rate due to the extensive difference among diagnostic criteria and definitions (Speroff & Fritz, 2011). Different studies reported a rate of 54-90% for the prevalence of PMS (Abdollahifard et al., 2013; Bakhshani et al., 2011; Braverman, 2007). The real cause of PMS is almost unknown, and theories in this regard include the increased aldosterone activity, elevated adrenal function, hyperprolactinemia, hypoglycemia, decreased levels of central dopamine and serotonin, decreased vitamin B6, and decreased essential fatty acids, of which the decreased levels of central dopamine and serotonin has been received more attention than other causes (Ghazi-Jahani, 2004; Sina, 1999; Ghazi-Jahani, 2007).

Many treatment protocols have been suggested because there is no specific pathophysiology for PMS. The suggested treatments are as follows: bromocriptine for breast congestion and pain, serotonin reuptake inhibitors (SSRIs) such as fluoxetine, antiprostaglandin medicines, GnRH analogues, vitamins, and herbal medicine. Currently, the most efficient treatment is the use of fluoxetine that has side effects, including insomnia, nervousness, and decreased libido (Ghazi-Jahani, 2004; Sina, 1999; Steiner et al., 2006; Ghazi-Jahani, 2007).

The overall goal of treating PMS is the adequate control of the symptoms in a way that the patient can function appropriately in the entire menstrual cycle (Speroff & Fritz, 2011; Kashani, 2010). There is no unique treatment for PMS (Braverman, 2007). The treatment approach is that all the patients with PMS should be followed up using treatments with least side effects (Baker, 2008). In this regard, treatment methods include medical therapy, the use of food supplements and vitamins, surgeries, and complementary alternative medicine (Ernest et al., 2011; Kim & Freeman, 2010; Ozgoli et al., 2011).

The use of vitamin is one of the most common treatment for every disease because they have fewer side effects than other medications, and they are also safe, cost-effective, and accessible. Furthermore, vitamins have been recommended as the benefit treatment that is safe, inexpensive, and effective in treatment of PMS (National Academy of Sciences, 1998).

Vitamin B1 (Thiamin) may reduce symptoms of PMS through the coenzyme functions in the metabolism of carbohydrates and main branch of amino acid that plays an important role in appearance of physical and mental symptoms of PMS. Vitamin B1 is the first water-soluble discovered vitamin. As it is effective in neural activity and muscle tonus in different body activities, including hematopoiesis, metabolism of carbohydrates, activities of the central nervous system and neuromuscular system, etc., it can be effective in this dysmenorrhea that is a disorder resulting from uterine muscular contraction. It has almost no side effect although long-term use of vitamin B1 may cause headaches and, sometimes, palpitations. However, extra vitamin B1 is excreted in the urine due to its water solubility (Wyatt et al., 1991; National Academy Press, 1998).

Vitamin B1 is used to treat varieties of physiological problems, such as nausea and vomiting; increase the quality of life; and decrease depression, fatigue, dysmenorrhea, muscle cramps, and anxiety in middle-aged women and women of reproductive age (McCormick, 1988).

Some studies have reported the therapeutic effect of vitamin B on mild and moderate depression, and also its positive results in problems related to women of reproductive age. No side effect has been reported in patients using vitamin B in different studies. (Wyatt et al., 1991; National Academy Press, 1998; McCormick, 1988; McCormick, 1997; Bayliss et al., 1984; Bailey, 1994; Singleton et al., 1995; Gerrits et al., 1997; Yaghmaei et al., 2005).

Certain studies conducted on determining the effect of vitamin B1 on problems related to women of reproductive age showed positive results. However, regarding the inadequate research on the effect of vitamin B1 on severity of the PMS symptoms, high prevalence of PMS, side effects and high cost of invasive methods, the increasing tendency to complementary medicine, and lack of research in this regard in Iran, this study was conducted to determine the effect of vitamin B1 on the PMS symptoms in students residing at dormitories of Jahrom University of Medical Sciences in 2013. The researcher chose vitamin therapy as a practical strategy to control severity of the PMS symptoms.

## 2. Materials and Methods

This clinical trial was performed on students residing at dormitories of Jahrom University of Medical Sciences. Samples were selected using purposive sampling method. The sample included 80 students who had mild to moderate PMS and met the inclusion criteria and resided at dormitories of Jahrom University of Medical Sciences. However, the sample size was estimated 100 students due to the possible dropout.

Duration of sampling was 8 months ending in Aug. 2013. The inclusion criteria were as follows: having regular menstrual cycles between 24 to 35 days, aged 18 to 30 years old, bleeding for 3 to 8 days, and being single. The exclusion criteria were as follows: suffering mental or physical diseases; using antidepressants, hormones, contraceptives (used to treat some diseases), vitamins, and herbal medications; exercising regularly; experiencing stress in the last three months; death of close relatives; marrying; and undergoing surgical operation.

**Figure 1:**
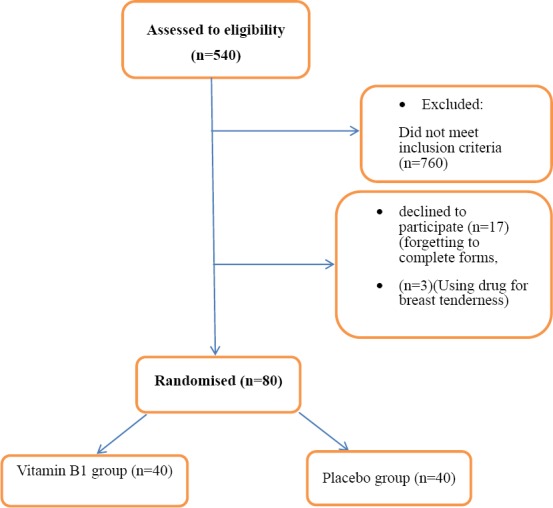
Selection of samples

The samples in this study were first selected purposively then were divided into two groups randomly using the Random Allocation software. In this regard, the researcher went to the dormitories of Jahrom University of Medical Sciences and gave a questionnaire about the inclusion criteria to the students. The students who had the inclusion criteria were given the PMS provisional diagnosis form. Then, those who had at least 5 symptoms were given Beck Depression Inventory. If the students were diagnosed to be normal and non-depressed based on their obtained score, they were selected as a research sample and requested to submit a written consent. Then, a demographics questionnaire was completed by the samples. The samples also completed the daily status record form for two consecutive cycles. The samples who exercised self-treatment was excluded (Figure 1).

The daily status record form included 20 symptoms of PMS, which were adopted from DSMIV criteria, as follows: tension, ambivalence, irritability, anxiety, depression, fatigue, headache, forgetfulness, palpitations, decreased libido, increased appetite, suicidal ideation, limb edema, breast tenderness, sleep disorder, Desire to eat sweets, flatulence, poor concentration, crying for no particular reason, and incompatibility. The students experiencing at least 5 symptoms 7 days before menstruation up to 4 days after menstruation and no symptoms in the rest of the cycle were diagnosed to have PMS. The samples marked the severity of their daily symptoms as none to severe (0-3). Content validity was used to measure the validity of the questionnaires. In this regard, the questionnaires were formulated on the basis of relevant books, articles, previous studies, corrective comments of specialists in obstetrics, gynecology, psychiatry, psychology, and pharmacology, and then given to 5 people of the study population and their comments were applied. To determine reliability of the questionnaires, the Cronbach’s alpha was used. In this regard, the questionnaires were given to 10 people of the study population, and the Cronbach’s alpha of 80% was obtained.

Finally, 100 students with inclusion criteria were divided into two groups with specific coding using the Random Allocation software and computerized table of random list. The first group consisted of 50 students using placebo, and the other group consisted of 50 students using vitamin B1. Two pills with identical appearance made by the advisor pharmacist were given to the placebo group (pills containing 100 mg of starch powder) and vitamin B1 group (pills containing 100 mg of vitamin B1 made by Tehran Daroupakhsh Co.) (European Commission. Health & Consumer Protection Directorate-General, 2011; Fao/Who expert consultation on human vitamin and mineral requirements, 2011). The samples used one pill in the morning and one pill at night during one week before menstruation (at the end of the luteal phase) when the symptoms were most severe for three consecutive cycles and, concurrently, completed the questionnaires through marking symptoms. Meanwhile, the samples were ensured of the lack of serious side effects of the pills and were requested to inform the researcher the incidence of any side effect or problem in order to be followed. To ensure recording of information by the samples, they were texted every week, and a short visit was held every two weeks.

The daily status record form was examined after three months of treatment, and physical and mental symptoms were measured separately before and after the treatment. Of the samples, 10 samples withdrew from the study, 3 samples were excluded from the study because they needed taking medicines for their severe breast tenderness, and 7 samples were also excluded from the study because they did not complete the questionnaires. In total, 20 samples were excluded from the study. The data collected form 80 samples (40 samples in the placebo group and 40 samples in vitamin B1 group) were analyzed using SPSS16 software.

Paired t-test was used to measure variations in severity of PMS in each group before and after the intervention, and the independent t-test was used to measure the difference in severity of PMS between the two groups. Moreover, the Chi-square test was used to determine the tendency to continue using pills, and Fisher’s exact test was used to determine the presence of side effects. The significance level for all tests was P < 0.05.

## 3. Results

Of the 540 students screened primarily, 100 students with inclusion criteria were given the daily status record form for two cycles and divided into two groups, 50 students received placebo, and 50 students received vitamin B1. The samples were visited every two weeks in order to check the course of taking pills and side effects. Finally, 20 samples were excluded from the study for different reasons, such as the failure to complete the questionnaires, the failure to use the pills at the right time, unwillingness to continue, and incidence of side effects. Therefore, the final analysis was performed on 80 samples. The demographic variables and severity of symptoms in each group were compared to one another before the treatment, and their homogeneity was proved (Table 1). Mean age of samples was 21 years. In most of the samples, the menstrual bleeding and menstrual cycle interval were reported as 5 days and 28-30 days, respectively.

**Table 1:** The comparison of the average of Age, Menarrche age, Interval of mensturation period and Duration of bleeding menses in two groups: Vitamin B1 group and control group

Group	Vitamin B1 group	Placebo group	P
	
	Average of score + standard deviation	Average of score + standard deviation	
**Age**	21.22 *±* 0.8	21.40 *±* 0.54	NS
**Menarrche age**	12.63 *±* 0.46	12.08 *±* 0.19	NS
**Interval of mensturation period**	28.39 *±* 0.77	29.08 *±* 0.33	NS
**duration of bleeding menses**	5.6 *±* 0.21	5.41 *±* 0.19	NS

The total mental and physical symptom in the two groups was the same. According to the daily status record form, there was no significant difference between placebo group and vitamin B1 group in terms of the mean total physical symptom before the treatment, and both groups were matched in this regard (Table 2).

**Table 2:** The comparison of the average of general (overall) severity of the symptoms of premenstrual syndrome before treatment in students of the dormitories of Jahrom University in 2013

Symptoms of PMS	Vitamin B1 group	Placebo group	P
	Average of score + standard deviation	Average of score + standard deviation	
**General (overall) severity of the symptoms of PMS**	47.49 ± 0.34	47.40 ± 0.54	NS
**Mental symptoms severity of PMS**	42.52 ± 0.02	42.59 ± 0.07	NS
**Physical symptoms severity of PMS**	34.19 ± 0.72	35.27 ± 0.98	NS

As shown in Tables 3, 4, and 5, the two groups were not significantly different from each other in terms of the severity of symptoms, and results of the treatment showed that mean overall severity in vitamin B1 group in the first month and the second month of the treatment was 23.45 and 15.40, respectively. In placebo group, mean overall severity in the first month and the second month of the treatment was 45.4 and 35.40, respectively. Mean reduction of overall severity after two months in vitamin B1 group and placebo group was 32.09% and 12%, respectively. Mean reduction of overall severity for physical and mental symptoms after three months in vitamin B1 group was 21.2% and 35.08%, respectively. The comparison of variations in severity of symptoms before and after the intervention of both groups together revealed that overall severity of symptoms after the intervention in both groups decreased significantly (P < 0.001). However, vitamin B1 decreased the symptoms significantly more than placebo (P < 0.0001), (Table 3).

**Table 3:** The comparison of the average of general (overall) severity of the symptoms of premenstrual syndrome before and after treatment in students of the dormitories of Jahrom University in 2013

Treatment stage	Vitamin B1 group	Placebo group	Result of test (Mann- Whitney)
	Average of score + standard deviation	Average of score + standard deviation	
**Before treatment**	47.49 *±* 0.34	47.40 *±* 0.54	NS
**After one month treatment**	23.45 *±* 0.83	45.40 *±* 0.76	0.0001< P
**After two months treatment**	15.40 *±* 0.52	35.40 *±* 0.76	0.0001< P
**The average of reduction of general severity of the symptoms after treatment**	32.09 *±* 0.73	12 *±* 0.49	0.0001< P
**Intra – group difference**	0.0001< P	0.001< P	
**(Wilcoxon test)**			

The average general severity of the symptoms of premenstrual syndrome has been reduced in Vitamin B1 group after treatment.

**Table 4:** The comparison of the average of mental symptoms severity of premenstrual syndrome before and after treatment in students of the dormitories of Jahrom University in 2013

Treatment stage	Vitamin B1 group	Placebo group	Result of test (Mann- Whitney)
	Average of score + standard deviation	Average of score + standard deviation	
**Before treatment**	42.52 *±* 0.02	42.59 *±* 0.07	NS
**After one month treatment**	19.40 *±* 0.63	42.51 *±* 0.94	0.0001< P
**After two months treatment**	7.44 *±* 0.63	42.51 *±* 0.94	0.0001< P
**The average of reduction of general severity of the symptoms after treatment**	35.08 *±* 0.50	0.08 *±* 0.46	0.0001< P
**Intra – group difference**	0.0001< P	0. 001< P	
**(Wilcoxon test)**			

The average mental symptoms severity of premenstrual syndrome has been reduced in Vitamin B1 group after treatment.

**Table 5:** The comparison of the average of physical symptoms severity of premenstrual syndrome before and after treatment in students of the dormitories of Jahrom University in 2013

Treatment stage	Vitamin B1 group	Placebo group	Result of test (Mann- Whitney)
	Average of score + standard deviation	Average of score + standard deviation	
**Before treatment**	34.19 *±* 0.72	35.27 *±* 0.98	NS
**After one month treatment**	19.40 *±* 0.63	33.51 *±* 0.94	0.0001< P
**After two months treatment**	12.99 *±* 0.63	30.51 *±* 0.94	0.0001< P
**The average of reduction of general severity of the symptoms after treatment**	21.20 *±* 0.63	4.76 *±* 0.46	0.0001< P
**Intra – group difference**	0.0001< P	0.001< P	
**(Wilcoxon test)**			

The average physical symptoms severity of premenstrual syndrome has been reduced in Vitamin B1 group after treatment.

The average of severity of mental symptoms of premenstrual syndrome in vitamin B1 group and control group was 7.44 ± 0.63 and 42.51 ± 0.94 respectively. Wilcoxon statistical test showed significant statistical difference in this respect between two groups (p < 0.0001) and in order to compare the groups (two by two), Mann–Whitney test showed significant statistical difference between two groups with (p < 0.0001), (Table 4).

The average of severity of physical symptoms of premenstrual syndrome in vitamin B1 group and control group was 12.99 ± 0.63 and 30.51 ± 0.94 respectively. Wilcoxon statistical test showed significant statistical difference in this respect between two groups (p < 0.0001) and in order to compare the groups (two by two), Mann–Whitney test showed significant difference between two groups with (p < 0.0001), (Table 5).

Maximum reduction of severity was observed in symptoms, including anxiety (96%), depression (80.4%), sleep disorder (80.24%), fatigue (73.88%), poor concentration (70.51%), tension (55.2%), incompatibility (39%), suicidal ideation (18%), headache (8.38%), flatulence (7.98%), and breast tenderness (7.33%). Minimum reduction of severity was observed in symptoms, including the decreased libido (0.94%), desire to eat sweets (2.42%), limb edema (2.6%), forgetfulness (3.44%), and palpitations (3.71%). The tendency to continue using pills in vitamin B1 group and placebo group was 95% and 12.4%, respectively, and their difference was significant (P < 0.05). Vitamin B1 was ineffective in the duration of menstruation and bleeding, and most of the samples, 98.3% in vitamin B1 group and 100% in placebo group, did not report any side effects.

The average of physical symptoms severity of premenstrual syndrome has been reduced in vitamin B1 group after treatment, and the comparison was significantly different.

The average of mental symptoms severity of premenstrual syndrome has been reduced in vitamin B1 group after treatment, and the comparison was significantly different.

## 4. Discussions

The results of this study showed that vitamin B1 reduced overall severity of symptoms and mental and physical symptoms of PMS in vitamin B1 group. There is no study was conducted on the effect of vitamin B1 on PMS in Iran, and there were few foreign studies in this regard. Therefore, comparing results of this study with those of other studies was limited. In their study on the comparison of the effect of vitamin B1 with that of ibuprofen, Zafari and Agha Mohammadi explained that vitamin B1 with an effect similar to that of Ibuprofen had fewer side effects and was more acceptable on treatment of primary dysmenorrhea (Agha mohammadi & Zafari, 2009). In Yaghmayi et al.’s study on the effect of combined vitamins B1 and B6 on leg cramps during pregnancy, samples in case and control groups received treatment with combined vitamins B1 and B6. After four weeks of follow-up, all the samples receiving treatment stated that combined vitamins B1 and B6 had reduced the number and severity of leg cramps (Yaghmaei et al., 2005). Moreover, some studies showed the effectiveness of vitamin B1 in decreasing depression, stress, anxiety, pain, fatigue, and sleep disorders; increasing the quality of life; and promoting blood circulation, metabolism of carbohydrates and aminoacids. As the above symptoms are also included in symptoms of PMS, a few studies in this regard are explained below (Wyatt et al., 1991; National Academy Press, 1998; McCormick, 1988; McCormick, 1997; Bayliss et al., 1984; Bailey, 1994; Singleton et al., 1995; Gerrits et al., 1997; Yaghmaei et al., 2005; Agha mohammadi & Zafari, 2009; Patricia et al., 2011; Bertone-Johnson et al., 2009; Gabriel, 2008).

Patricia Chocano et al.’s study on the effect of a diet containing vitamin B1 on the incidence of PMS showed that receiving vitamin B1 and riboflavin from food reduced the risk of PMS. Gabriel conducted a study on vitamin B complex and its effects, and found that vitamin B1 was effective in releasing energy and improving blood circulation and digestion. Moreover, Gabriel showed that 30 mg of thiamine (vitamin B1) reduced skeletal muscle cramps, promoted perfusion to the heart, kidneys, liver, and brain, and was effective in metabolism of energy and nervous system functioning. Results of the present study agreed with those of Patricia Chocano et al.’s study and Gabriel’s study. As revealed by the present study, vitamin B1 could reduce all symptoms of PMS significantly (Patricia et al., 2011; Gabriel, 2008).

Although the exact cause of PMS has not been known so far, the sexual hormones and central neurotransmitters are discussed more than other possible causes. Most symptoms of PMS are similar to those of reduced serotonin neurotransmitters, and thus, the first-line treatment to cure PMS is the use of methods with the mechanism of serotonin reuptake inhibitors (Ghazi-Jahani, 2007).

As a coenzyme, vitamin B1 may reduce symptoms of PMS through affecting the performance of coenzymes in the metabolism of carbohydrates, lipids, protein and main branch of amino acid. Vitamin B is effective in energy metabolism, activities of the central nervous system, hematopoiesis, metabolism of carbohydrates, activities of neuromuscular system, muscle and cardiovascular tonus, promotion of blood circulation, and consequently, excretion of toxins from the body (Yaghmaei et al., 2005; Agha Mohammadi & Zafari, 2009).

Gabriel pointed out that vitamin B1 could increase endorphin secretion and, subsequently, cause relaxation and stimulate sleeping through affecting the brain and liver and releasing ATP, and it could also promote cardiovascular system functioning. Thiamine affects the urinary tract, erythrocyte transketolase of blood circulation, blood lactate and pyruvate, and neurological changes (Gabriel, 2008). The role of alcohol in vitamin B1 reuptake is of special importance, and alcoholics are more likely to suffer psychological disorders and PMS. Nutritionists recommend using 1.1 mg of vitamin B1 per day in adult women for promoting energy level, and this amount of vitamin B1 should be much more for pregnant and lactating women. Therefore, the decrease in symptoms of PMS, especially mental symptoms, is justifiable (Bertone-Johnson et al., 2009).

Hemostasis and hormonal imbalance are important etiologies of PMS (Steiner et al., 2006). In this respect, reduction of symptoms, such as flatulence, headache, breast pain, and palpitations with vitamin B1 is also justifiable. Vitamin B1 simulates the central nervous system and elevates mood, and consequently, causes deep relaxation and decreases mental pressure and tension. Therefore, the decrease in symptoms of PMS, especially mental symptoms, is reasonable.

Similar to Patricia Chocano et al.’s study, the present study showed that vitamin B1 had no side effects. In other words, vitamin B1 was effective in treatment of PMS without any side effects. In this respect, regarding the above advantage of vitamin B1, which was proved in this study, and prospective control of recording symptoms for 3 months, results of the present study are reliable.

## 5. Conclusion

This study, which was performed for the first time in Iran, revealed that using vitamin B1 in luteal phase could reduce overall severity of physical and mental symptoms of PMS needless of using it in the entire menstrual cycle. Furthermore, vitamin B1 has no side effect and does not change menstrual bleeding pattern. Therefore, vitamin B1 is recommended for treatment of PMS.

Moreover, further studies are recommended to obtain enough evidence for injection of vitamin B1 and its different dosages as a safe and effective medication for patients who do not desire to or cannot undergo invasive treatments.

Furthermore, the starch powder was used as the placebo in this study. Given the effect of carbohydrates on alleviating PMS, further studies are suggested to use neutral powders.
